# Microbiome Dynamics Associated With the Atacama Flowering Desert

**DOI:** 10.3389/fmicb.2019.03160

**Published:** 2020-01-22

**Authors:** Juan Pablo Araya, Máximo González, Massimiliano Cardinale, Sylvia Schnell, Alexandra Stoll

**Affiliations:** ^1^Centro de Estudios Avanzados en Zonas Áridas, La Serena, Chile; ^2^Department of Biological and Environmental Sciences and Technologies, University of Salento, Lecce, Italy; ^3^Institute of Applied Microbiology, Justus Liebig University Giessen, Giessen, Germany; ^4^Instituto de Investigación Multidisciplinario en Ciencia y Tecnología, Universidad de La Serena, La Serena, Chile

**Keywords:** *Cistanthe longiscapa*, Atacama Desert, desert bloom, precipitation gradient, rhizosphere community, core microbiome, co-occurrence patterns

## Abstract

In a desert, plants as holobionts quickly respond to resource pulses like precipitation. However, little is known on how environment and plants modulate the rhizosphere-associated microbiome. As a model species to represent the Atacama Desert bloom, *Cistanthe longiscapa* (*Montiaceae* family) was selected to study the influence of abiotic and biotic environment on the diversity and structure of the microbiota associated to its rhizosphere. We analyzed the rhizosphere and soil microbiome along a North-South precipitation gradient and between a dry and rainy year by using Illumina high−throughput sequencing of 16S rRNA gene fragments and ITS2 regions for prokaryotes and fungi, respectively. In the rhizosphere of *C. longiscapa* the microbiota clearly differs in composition and structure from the surrounding bulk soil. The fungal and bacterial communities respond differently to environmental conditions. The diversity and richness of fungal OTUs were negatively correlated with aridity, as predicted. The community structure was predominantly influenced by other soil characteristics (pH, organic matter content) but not by aridity. In contrast, diversity, composition, and structure of the bacterial community were not influenced by aridity or any other evaluated soil parameter. These findings coincide with the identification of mainly site-specific microbial communities, not shared along the sites. These local communities contain a group of OTUs, which are exclusive to the rhizosphere of each site and presumably vertically inherited as seed endophytes. Their ecological functions and dispersal mechanisms remain unclear. The analysis of co-occurrence patterns highlights the strong effect of the desert habitat over the soil- and rhizosphere-microbiome. The site-independent enrichment of only a small bacterial cluster consistently associated with the rhizosphere of *C. longiscapa* further supports this conclusion. In a rainy year, the rhizosphere microbiota significantly differed from bulk and bare soil, whereas in a dry year, the community structure of the former rhizosphere approximates to the one found in the bulk. In the context of plant–microbe interactions in desert environments, our study contributes new insights into the importance of aridity in microbial community structure and composition, discovering the influence of other soil parameters in this complex dynamic network, which needs further to be investigated.

## Introduction

**FIGURE 1:**
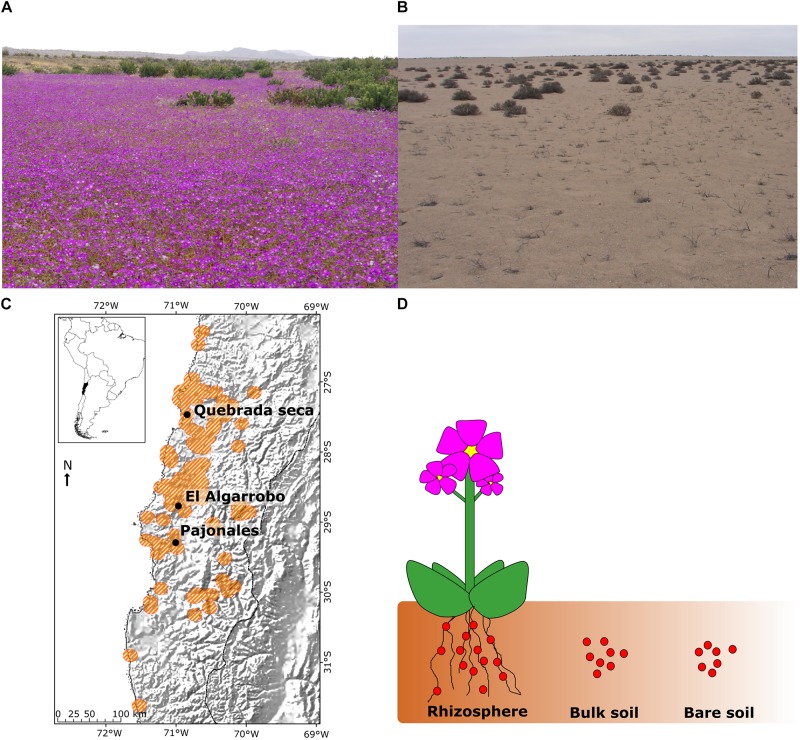
Natural habitat of *Cistanthe longiscapa*, sampling sites and sampling strategy per site. **(A)** Photograph of *C. longiscapa* population in a rainy year; **(B)** Photograph of *C. longiscapa* population in a dry year; **(C)** Geographical localization of sampling sites in Quebrada Seca, El Algarrobo, and Pajonales; **(D)** Schematic illustration of sample types from each site: starting from a *C. longiscapa* individual three sample types were distinguished: Rhizosphere, Bulk soil, and Bare soil^∗^. ^∗^Bare soil was only collected at site Quebrada Seca, for comparison of rainy and dry year.

The Atacama Desert in Chile is one of the most arid and ancient deserts on the planet (Hartley et al., 2005; Bull et al., 2016), where small and irregular water pulses allow the survival of an adapted plant cover composed primarily of annual species (Noy-Meir, 1973). During rainy periods (years involving the “El Niño” phenomenon), the increased water availability can cause a desert bloom event, where multiple annual plants quickly respond by germinating, emerging and flowering, varying in their time of growth, permanence and abundance. During this event, the desert landscapes between 27 and 29°S are covered with intense pink, white, yellow and purple colors, transforming the Atacama Desert into an exuberant garden between the months of August and October. *Cistanthe longiscapa* (Barnéoud) Carolin ex Hershkovitz (*Montiaceae* family), a native annual plant, is one of the most abundant species when the peak of this phenomenon is reached. The typical life cycle of *C. longiscapa* lasts only about 2 months, time span in which the plant establishes all biotic interactions, e.g., with soil microbiota (Armesto et al., 1993; Vidiella et al., 1999) (Figure 1A).

The desert bloom significantly increases the primary production and causes subsequent biological interactions between herbivores and predators, changing the structure and functioning of the ecosystem (Holmgren et al., 2006). Although plant cover is known to be important for ecological interactions in desert soil, few studies have investigated microbial ecology, community structure and their interactions with plants, the latter of which can improve the fitness of the plant under conditions of limited environmental resources (Lugtenberg and Kamilova, 2009; Bresson et al., 2013).

In arid environments, microbial communities structure depends on (1) environmental factors, where aridity degree negatively correlates with diversity and abundance of taxa, favoring those that are better adapted (Maestre et al., 2015); (2) seasonal periods, rainy or dry (Coleman et al., 2016; Postma et al., 2016; Taketani et al., 2017); (3) plant species, where the relative abundance of certain microbial taxa depends on their host-specificity (Fonseca-García et al., 2016); and (4) soil physico-chemical properties (Coleman et al., 2016; Na et al., 2018). Jorquera et al. (2016) reported that microbial communities in the rhizosphere of perennial Atacama Desert plants vary according to both environment and plant. Additionally, plants not only modulate rhizospheric communities, but maintains a “core microbiome” composed by species which are enriched in the rhizosphere, regardless the sampling location (Lundberg et al., 2012; Vandenkoornhuyse et al., 2015) or plant development stage (Pfeiffer et al., 2017). This “core microbiome” represents a small fraction of the total diversity in the rhizosphere but fulfils important ecosystem functions (Colin et al., 2017; Pfeiffer et al., 2017).

The adaptation of *C. longiscapa*, locally known as “*Pata de Guanaco*” to such a hostile habitat converts the plant into an interesting model species to investigate on drivers of change and dynamics of plant-associated microbiomes in hyperarid soils. In this sense, such extreme environments are poorly studied, which is the reason why we set up the following hypotheses: (1) the diversity of the microbiota associated to *C. longiscapa* rhizosphere is negatively correlated with aridity (selection by abiotic environment), both in spatial (same season, different sites) and temporal scale (same site, different years); (2) a group of microorganisms specifically associated to the rhizosphere of *C. longiscapa* exists, independently from the locality (selection by biotic environment). On the other hand, as a null hypothesis, we would find no significant correlation between microbial community composition and plant populations, reflecting that perhaps the established gradient does not affect the soil microbial community. Then the plant would shape the root-associated microbiome independently of the sampling site. The results of our study will help to elucidate the dynamics of the microbial communities associated to annual plants in extremely arid environments.

## Materials and Methods

### Sampling Strategy and Soil Analyses

Three populations of *C. longiscapa* were investigated in three different locations: Quebrada Seca (QS15), El Algarrobo (EA15), and Pajonales (PA15), which are representative of the distribution area (25–31 °S) along a latitudinal gradient (increase in rainfall from north to south) (Figure 1C and Supplementary Table S1).

Sampling was conducted during the desert bloom in September 2015. Two sample types, rhizosphere (R) and bulk soil (B) were collected separately (Figure 1D). The rhizosphere area was sampled by carefully digging about 30 cm deep around the plant (and 30 cm in diameter around the plant). The soil near the roots was easily removed by gently shaking and the fraction of soil firmly attached to the roots was collected (with the roots) as rhizospheric soil. To cover the representativeness of each sampling site, three independent replicates were collected. Each replicate consisted in the rhizosphere of 10 *C. longiscapa* individuals of similar size at flowering state (≥ 3 flowers), covering an approximate area of 10,000 m^2^ per location. At each location, additional soil samples were collected to determine the concentration (mg/kg) of N, P, K, organic matter (OM%) and pH, as service realized by Technological Centre of Soil and Cultivation [Centro Tecnológico de Suelo y Cultivo (CTSyC)], Talca, Chile.

The influence of a rainy or dry year (absence of the El Niño phenomenon) on the microbiota associated with *C. longiscapa* was studied at Quebrada Seca (QS15 and QS16), which represents the highest degree of aridity. In September of two consecutive years (2015 – rainy, 2016 – dry), rhizosphere (R), bulk soil (B), and bare soil (BS) (absence of vegetation) samples were collected, in three independent replicates each (Figures 1B,D). The rhizosphere sample from 2016 was composed of soil corresponding to the former rhizospheric zone of dead *C. longiscapa* individuals (termed as “dormant”). All samples were transferred to sterile 50 mL tubes and maintained at −20°C until analysis.

### DNA Extraction and Illumina Sequencing

Soil metagenomic DNA was extracted according to Devi et al. (2015). Prokaryotic 16S ribosomal RNA gene (Lundberg et al., 2013) and fungal ITS2 region (Op De Beeck et al., 2014; Coleman et al., 2016) were amplified and sequenced on the Illumina MiSeq platform by Macrogen, Inc. (Seoul, South Korea).

The sequence analysis was performed using QIIME 1.9 (Caporaso et al., 2010). The sequences were filtered by length (200 to 1000 bp) and quality (score threshold = 25), and chimeric sequences were removed using Vsearch (Rognes et al., 2016). ITSx v.1.0.11 was used to extract fungal ITS sequences (Bengtsson-Palme et al., 2013). Retained sequences were grouped into operational taxonomic units (OTUs) at 97% sequence similarity using the uclust method and the SILVA (Quast et al., 2013) and UNITE (Abarenkov et al., 2010) databases for prokaryotes and fungi, respectively^1^. A representative sequence was selected for the identification of each OTU. All OTUs with less than 20 sequences were removed, as well as mitochondrial and chloroplast OTUs. The sequences were deposited in the NCBI database^2^ in four BioProjects to facilitate the download of the information separately. In the BioProjects PRJNA561568 and PRJNA561410 there are the libraries for analyses between sites, for fungi and bacteria, respectively. In the PRJNA561612 and PRJNA561600 BioProjects there are the libraries for analyses between rainy and dry year for fungi and bacteria, respectively.

### Alpha- and Beta-Diversity

All analyses were performed identically for fungi and prokaryotes. Taxa relative abundances were displayed in percentage bar graphs. The relative abundance of phyla was statistically compared between locations (ANOVA, Tukey *post hoc* test at *p* < 0.05) and between rhizosphere and bulk soil in each location (Student’s *t*-test). Subsequently, the data were rarefied to calculate alpha- and beta-diversity. Alpha-diversity was estimated using Shannon and Chao1 indices. Statistical differences were tested with ANOVA followed by Tukey HSD *post hoc* test. To assess similarity between communities (beta-diversity), weighted UniFrac distances were calculated and visualized using principal coordinates analysis (PCoA) (Na et al., 2017) and the significance of each factor (location, sample type, N, P, K, organic matter, and pH) was tested with ADONIS, as implemented in QIIME 1.9 (McArdle and Anderson, 2001).

The “rhizosphere-specific” community for each location was identified, according to the following criteria: (1) OTU present in the three rhizosphere replicates, (2) OTU with > 20 sequences among the three rhizosphere replicates, and (3) average number of sequences for an OTU in rhizosphere ≥ 3 times compared with the bulk soil (fold change ≥ 3); significant differences between OTUs for soil type in each location were calculated with Student’s *t*-test at *p* < 0.05. The “core microbiome” among “rhizosphere-specific” OTUs that were present in all locations was visualized as a Venn diagram^3^.

### Analysis of Co-occurrence Network

To detect significant microbial relationships and consortia of taxa in the *C. longiscapa* rhizosphere and bulk soil, a co-occurrence analysis was performed with the Co-occurrence Network inference software (CoNet; Faust and Raes, 2016). Not-normalized data were used to reduce compositional effect (Berry and Widder, 2014). After merging fungal and bacterial OTU tables, OTUs with < 500 reads were deleted (Berry and Widder, 2014). Pairwise scores were computed for the following similarity measures: Bray–Curtis, Kullback–Leibler, Pearson and Spearman. A threshold of 2,000 edges for the initial network was used. For each measure and edge, 100 permutations (with renormalization for correlation measures and row-shuffling resampling) and bootstrap scores were generated. Unstable edges (outside the 2.5–97.5 percentiles of the bootstrap distribution) were removed. The single *p*-values generated by the four methods were merged using the Brown’s method (Volterra, 1926). After false discovery rate (FDR)-correction (Benjamini and Hochberg, 1995), edges with *p-*values below 0.05 were kept. Considering that Bray–Curtis and Kullback–Leibler distances are robust to compositionality, while Pearson and Spearman are more sensitive (Faust and Raes, 2016), only edges supported by ≥ three similarity measures were retained.

## Results

### Microbiome Dynamics Along the Latitudinal Gradient

#### Taxonomic Composition of the Microbiota

A total of 716 fungal and 3,293 prokaryotic OTUs were obtained (Supplementary Table S2). The sequencing depth captured most of the diversity present in the samples (Supplementary Figures S1a,b), which increased from North to South for fungi but not for prokaryotes (Supplementary Figures S1c,d).

The assignment of fungal OTUs revealed 8 phyla, 26 classes, 57 orders, 114 families, and 178 genera. Significantly, the most abundant phylum was *Ascomycota* (84.9 ± 13.1%, ANOVA, *p* < 0.05, Tukey *post hoc* test), no significant differences were detected between the phyla *Basidiomycota* (8.4 ± 11.17%), *Mortierellomycota* (1 ± 1.9%) and *Chytridiomycota* (0.8 ± 1.4%) (Supplementary Figure S2a), representing altogether 95.1% of the total reads over all samples. At each location, community composition at phylum level was different between rhizosphere and bulk soil. In Quebrada Seca and El Algarrobo, the rhizosphere was colonized almost exclusively by *Ascomycota* (97.3 and 98.5%, *t*-test FDR-corrected *p* < 0.05 and <0.001 respectively), while in Pajonales also *Basidiomycota* were abundant (12.1%, *t*-test FDR *p* < 0.05) (Supplementary Figure S2b).

For prokaryotes, 27 phyla, 83 classes, 168 orders, 295 families, and 588 genera were identified. *Actinobacteria* (43.4 ± 10.4%) and *Proteobacteria* (20.7 ± 5.1%) were significantly most abundant (ANOVA, *p* < 0.01, Tukey *post hoc* test), followed by *Chloroflexi* (8.9 ± 2.5%), *Firmicutes* (6.9 ± 5.1%), *Bacteroidetes* (6.5 ± 4.5%), *Acidobacteria* (4.5 ± 4.6%), *Planctomycetes* (3.5 ± 1.3%) and *Gemmatimonadetes* (2.1 ± 1.13%) (no significant differences were detected between these phyla), which represented together more than 95% of the total reads over all samples (Supplementary Figure S2c). Locations with a higher degree of aridity (Quebrada Seca and El Algarrobo) differed in community composition between rhizosphere and bulk soil, where *Proteobacteria* and *Bacteroidetes* were significantly increased in the rhizosphere of Quebrada Seca (*t*-test FDR *p* < 0.05), while *Actinobacteria* and *Firmicutes* were significantly enriched in the rhizosphere of El Algarrobo (*t*-test *p* < 0.05), whereas Pajonales exhibited subtle variations between the soil habitats (Supplementary Figure S2d).

Archaeal phyla were detected at low abundance (*Euryarchaeota*, 0.03%; *Thaumarchaeota*, 0.01%). 1.26% was not identified.

#### Diversity and Structure of the Rhizosphere Microbiota

**FIGURE 2:**
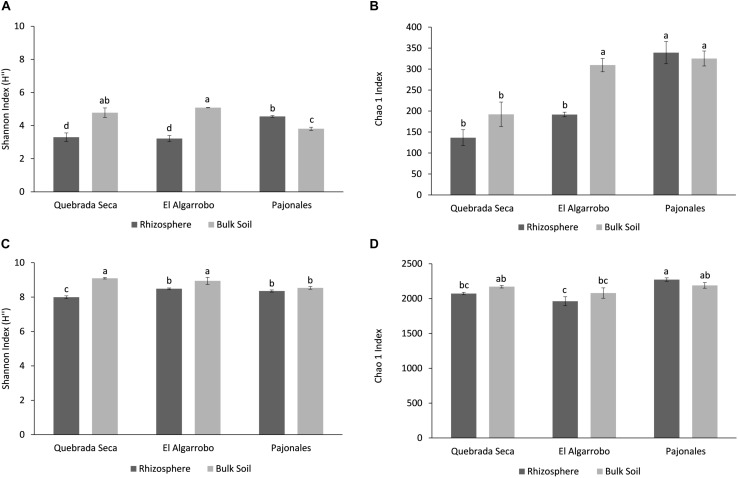
Alpha-diversity analysis per locality and sample type (rhizosphere and bulk soil). **(A)** Shannon Index for fungi; **(B)** Chao1 Index for fungi; **(C)** Shannon Index for prokaryotes; **(D)** Chao1 Index for prokaryotes. Different letters above the bars indicate statistically different means (Tukey test *p* < 0.05).

Alpha-diversity metrics were compared between rhizosphere and bulk soil in all locations, metrics were greater for Prokaryotes than for Fungi (Figure 2). The fungal Shannon diversity index in the rhizosphere of Quebrada Seca and El Algarrobo was significantly lower than in the corresponding bulk soil, while it was greater in Pajonales (ANOVA, *p* < 0.001, Tukey *post hoc* test) (Figure 2A). Chao1 richness estimator showed significant differences only between rhizosphere and bulk soil in El Algarrobo; however, it tended to increase with humidity (ANOVA, *p* < 0.001, Tukey *post hoc* test) (Figure 2B). For prokaryotes, the Shannon index significantly differed between rhizosphere and bulk soil of Quebrada Seca and El Algarrobo (ANOVA, *p* < 0.001, Tukey *post hoc* test) (Figure 2C). In contrast, Chao1 index did not differ significantly (ANOVA, *p* < 0.001, Tukey *post hoc* test) (Figure 2D).

**FIGURE 3:**
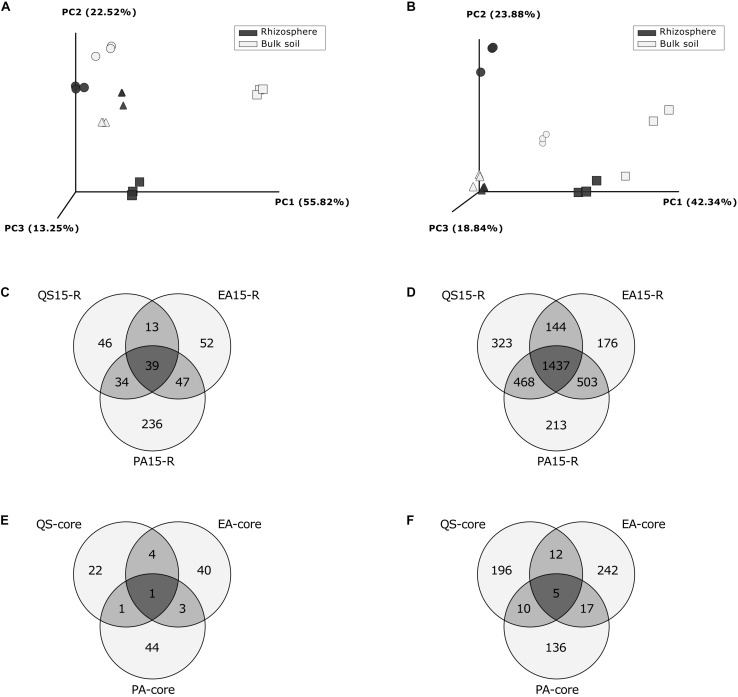
Beta-diversity analysis and Venn diagrams: comparison between localities. Principal coordinates analysis (PCoA) based on weighted UniFrac Distances per locality and sample type, for fungi **(A)** and for prokaryotes **(B)**; symbols represent localities: Circles: Quebrada Seca, Squares: El Algarrobo, Triangles: Pajonales; **(C)** Venn diagram for fungal OTUs registered in rhizosphere per locality; **(D)** Venn diagram for prokaryotic OTUs registered in rhizosphere per locality; **(E)** Venn diagram for rhizosphere specific fungal OTUs (fold change ≥ 3, between Rhizosphere and Bulk soil OTUs); **(F)** Venn diagram for rhizosphere specific prokaryotic OTUs (fold change > 3, between Rhizosphere and Bulk soil). QS, Quebrada Seca; EA, El Algarrobo; PA, Pajonales.

Weighted UniFrac distances showed a clear difference between rhizosphere and bulk soil community structures, at each location for both Fungi and Prokaryotes, except for the prokaryotic community in Pajonales, where difference was less pronounced (Figures 3A,B). At a macroecological scale (gradient), both the fungal and the prokaryotic community varied significantly among the three locations (ADONIS factor “site”: Fungi, *R*^2^ = 0.557, *p* = 0.001, Supplementary Table S3; Prokaryotes, *R*^2^ = 0.58, *p* = 0.001, Supplementary Table S4). At the same scale, the “sample type” had no influence on the microbiota structure (ADONIS, *p* > 0.1 for Fungi and Prokaryotes (Figures 3A,B and Supplementary Tables S5, S6).

Considering the other evaluated environmental parameters, the type of plant population, soil texture, phosphate content, nitrogen content, potassium content, organic matter and pH significantly affected the structure of the fungal and prokaryotic community (Table 1).

**TABLE 1:** Significance of the evaluated environmental parameters on the structure of the soil microbiota, assessed by ADONIS test, with 999 permutations.

**Environmental factor**	**Fungi**		**Prokaryotes**	
	***R*^2^**	***p***	***R*^2^**	***p***
Population type	0.41	0.001	0.37	0.001
Soil texture	0.27	0.005	0.22	0.005
Nitrogen	0.30	0.002	0.35	0.027
Phosphor	0.31	0.001	0.24	0.002
Potassium	0.15	0.05	0.26	0.003
Organic matter	0.38	0.001	0.29	0.002
pH	0.39	0.001	0.30	0.002

*NS, not significant (*p* > 0.05).*

#### “Rhizosphere-Specific” OTUs and Core Microbiome

Overall, 467 fungal and 3,264 prokaryotic OTUs were identified in the rhizosphere samples, 39 and 1,437, respectively, were shared between all locations (Figures 3C,D). At each location the unique and shared OTUs number of rhizosphere and bulk soil samples were identified for fungi and prokaryotes (Supplementary Figure S3). Additionally, at each location, a rhizosphere specific microbial community and rhizosphere-exclusive OTUs were detected (Supplementary Table S7). The number of rhizosphere specific OTUs for both, fungi and prokaryotes, increased along the precipitation gradient, except for prokaryotes in Pajonales (Figures 3E,F). In Quebrada Seca, 28 fungal “rhizosphere-specific” OTUs were identified, of which 6 OTUs were only present in the rhizosphere sample type and not in the bulk soil (Supplementary Table S8); 48 “rhizosphere-specific” OTUs were identified in El Algarrobo, of which 31 OTUs were only present in the rhizosphere (Supplementary Table S9); and 49 “rhizosphere-specific” OTUs were identified in Pajonales, of which 12 OTUs were only present in the rhizosphere (Supplementary Table S10). As for prokaryotes in Quebrada Seca, 223 “rhizosphere-specific” OTUs were identified, of which 44 OTUs were only present in the rhizosphere sample type and not in the bulk soil (Supplementary Table S11); 276 “rhizosphere-specific” OTUs were identified in El Algarrobo, of which 13 OTUs were only present in the rhizosphere (Supplementary Table S12) and 168 “rhizosphere-specific” OTUs were identified in Pajonales, of which 4 OTUs were only present in the rhizosphere (Supplementary Table S13).

The core microbiome in the rhizosphere of *C. longiscapa* was composed off 6 OTUs, corresponding to: *Fusarium concentricum* for fungi (Figure 3E and Supplementary Table S14) and *Pseudoxanthomonas* sp., *Ensifer* sp., *Mesorhizobium* sp., *Nocardioides* sp. as well as one OTU from the family *Comamonadaceae* for prokaryotes (Figure 3F and Supplementary Table S15). The abundance of these OTUs between locations was not uniform (Supplementary Figure S4). While *Fusarium concentricum* was 650 times and *Pseudoxanthomonas* sp. 12 times more abundant in Quebrada Seca compared to the other locations, *Nocardioides* sp. and *Ensifer* sp. were 68 and 4 times more abundant in the rhizosphere of Pajonales, respectively (ANOVA, *p* < 0.05, Tukey *post hoc* test) (Supplementary Tables S14, S15).

In addition, OTUs exclusive of the bulk soil were also detected in each locality. However, none of the 269 OTUs for fungi and 392 OTUs for prokaryotes was shared among the three locations (Supplementary Figures S5a,b).

#### Analysis of Co-occurrence Network

**FIGURE 4:**
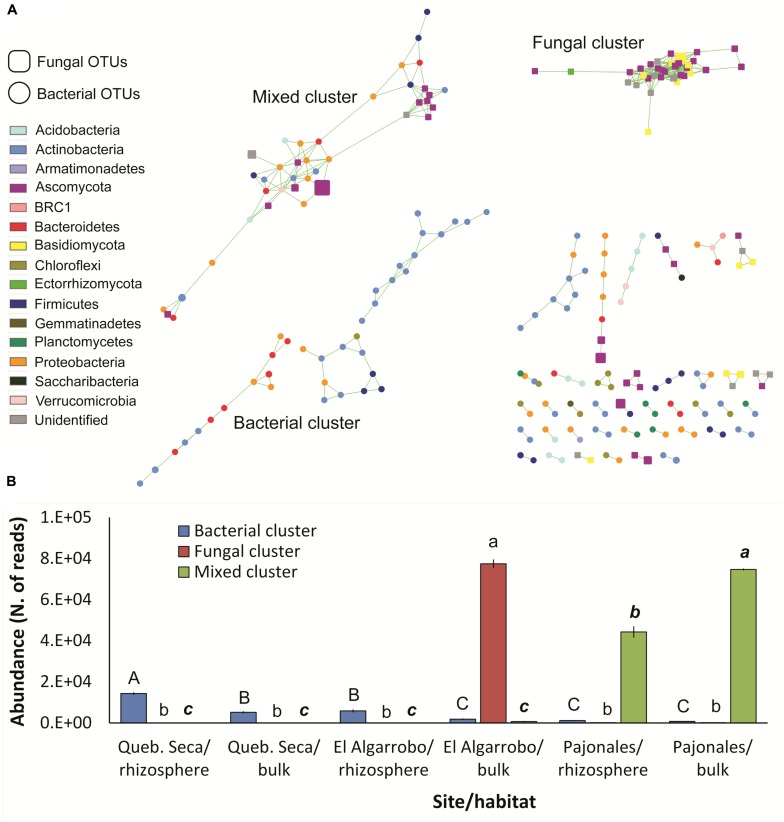
Network analysis of the bacterial-fungal microbiome associated to *C. longiscapa* during the Atacama flowering desert event. **(A)** Bacterial and fungal OTUs (round and squared nodes, respectively) are correlated to each other if connected by a green line. Node size indicates the respective mean OTU abundance. Node color indicates the taxonomic affiliation. The three main clusters detected are labeled. **(B)** Distribution of the three clusters across the three sites and the two habitats (rhizosphere/bulk). Different letters indicates significantly different means for each cluster (Tukey test, *p* < 0.05).

The network was composed by 149 bacterial and 70 fungal OTUs having at least one significant correlation. Total correlations were 409. Three consortia of correlated OTUs were detected: one composed only by bacteria, one only by fungi and one mixed (Figure 4A). These three clusters showed significantly different and complementary distributions across the three sites of the gradient and the two habitats (ANOVA, *p* < 0.001): the bacterial cluster, dominated by *Actinobacteria*, followed the arid gradient, being more abundant in the driest site and decreasing southwards; in each site, it was more abundant in the rhizosphere, although not significantly in Pajonales (Figure 4B). The fungal cluster was specific of the bulk soil in El Algarrobo (Figure 4B). The mixed cluster, the most diverse with 7 Phyla and 13 classes (Supplementary Table S16), occurred only in Pajonales and was significantly more abundant in the bulk soil (Figure 4B).

### Microbiome Dynamics Between a Rainy and a Dry Year

#### Taxonomic Composition of the Microbial Community

A total of 469 and 3,029 fungal and prokaryotic OTUs were obtained, respectively (Supplementary Table S17); the sequencing depth captured the diversity present in all the samples (Supplementary Figures S6a,b). A lower diversity of fungi was observed for the rainy year (Supplementary Figure S6c), no differences were observed for prokaryotes (Supplementary Figure S6d).

For fungi, 7 phyla, 30 classes, 66 orders, 132 families, and 201 genera were identified. Significantly, the most abundant phylum was *Ascomycota* (85.2 ± 7.8%, ANOVA, *p* < 0.05, Tukey *post hoc* test), no significant differences were detected between the phyla *Basidiomycota* (4.6 ± 3.9%), *Mortierellomycota* (1.5 ± 1.7%) and *Chytridiomycota* (1 ± 1.1%), representing ∼92.3% of the total relative abundance of phyla among all samples (Supplementary Figure S7a). The community assemblage differed between the sample types (rhizosphere, bulk soil, and bare soil) in each year, with fluctuating fungal populations observed between both years. In the rhizosphere, the phylum *Ascomycota* exhibited significantly the highest abundance, with 97.3% (± 1.4) in the rainy year (2015), decreasing to 80.9% (± 8.2) in the dry year (2016) (ANOVA, *p* < 0.05, Tukey *post hoc* test). In the dry year, although other phyla increased their relative abundances, such as *Basidiomycota* (11.6%), *Mortierellomycota* (2%), *Chytridiomycota* (1%) and unidentified (4.2%) phyla, these differences between years were not significant (ANOVA, *p* < 0.05, Tukey *post hoc* test). The rhizospheric community during the dry year was highly similar to the structure of the bulk soil community from both years. Bare soil samples (not vegetated soil) showed no variation between 2015 and 2016 (Supplementary Figure S7b).

For prokaryotes, 26 phyla, 81 classes, 158 orders, 288 families, and 587 genera were identified. The phyla with the significant highest relative abundances were *Actinobacteria* (37.1% ± 8.2%), *Proteobacteria* (25.2 ± 12.9%) (ANOVA, *p* < 0.05, Tukey *post hoc* test), no significant differences were detected between the phyla *Chloroflexi* (11.2 ± 5.4%), *Bacteroidetes* (6.5 ± 4.1%), *Firmicutes* (6.3 ± 5.0%), *Planctomycetes* (3.9 ± 1.4%), *Gemmatimonadetes* (3.1 ± 1.2%) and *Acidobacteria* (2.6 ± 1.5%), representing together more than 95% of the total relative abundance over all samples (Supplementary Figure S7c). The archaea *Euryarchaeota* (0.17 ± 0.24%) and *Thaumarchaeota* (0.01 ± 0.006%) exhibited low relative abundances. Similar as fungi, the prokaryotic community of the rhizosphere varied between the rainy and the dry year. The most abundant phylum during the rainy year in the rhizosphere was *Actinobacteria* (36.3 ± 5.2%), with no large fluctuations when comparing between soil types or years (ANOVA, *p* < 0.05, Tukey *post hoc* test). Other abundant phyla of the rhizosphere during the rainy year, like *Proteobacteria* (29.4 ± 4.1%) showed no significant difference between rhizosphere samples in both years, whereas *Bacteroidetes* (14.4 ± 0.7%), decreased significantly in relative abundance during the dry year (ANOVA, *p* < 0.05, Tukey *post hoc* test). In addition, the bacterial rhizosphere community in the dry year tended to be similar to its corresponding bulk soil sample (QS16-B). The bare soil community also varied between both years, as *Proteobacteria* considerably increased in relative abundance during the dry year, displacing, e.g., *Actinobacteria* and *Chloroflexi* (Supplementary Figure S7d).

#### Diversity and Structure of the Microbial Community Associated to the Rhizosphere

**FIGURE 5:**
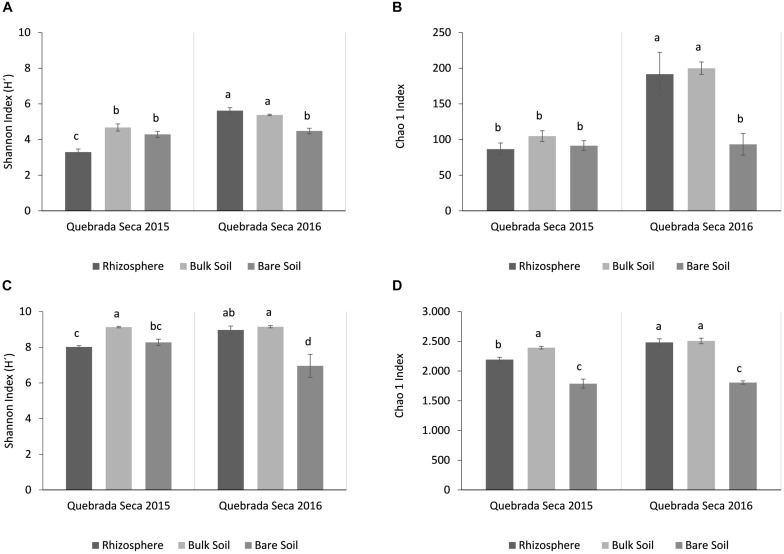
Alpha-diversity analysis per year and sample type (rhizosphere, bulk soil, bare soil). **(A)** Shannon Index for fungi; **(B)** Chao1 Index for fungi; **(C)** Shannon Index for prokaryotes; **(D)** Chao1 Index for prokaryotes. Different letters above the bars indicate statistically different means (Tukey test, *p* < 0.05).

The fungal diversity in the active rhizosphere (2015) was lower compared to the corresponding bulk and bare soils (Figure 5A), as well as to all soil types from the dry year. The highest richness (Chao1) was found in the dormant rhizosphere and bulk soil of the dry year (Figure 5B) (ANOVA, *p* < 0.05, Tukey *post hoc* test).

Similar to fungi, the active rhizosphere of the rainy year showed a low diversity of prokaryotes. However, the lowest prokaryotic diversity was detected in the bare soil of the dry year, which also comprised the lowest species richness (Figures 5C,D). Again, rhizosphere and bulk soil of the dry year registered the highest Chao1 values (ANOVA, *p* < 0.05, Tukey *post hoc* test).

**FIGURE 6:**
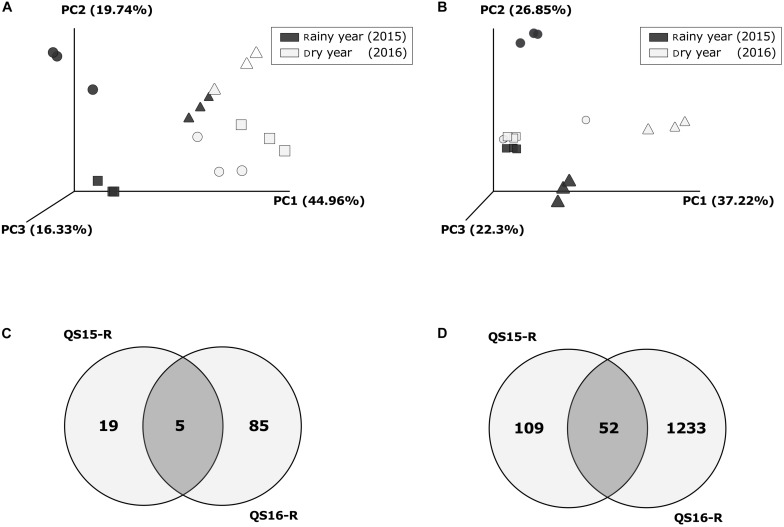
Beta-diversity analysis and Venn diagrams: comparison between years at Quebrada Seca (QS). Principal Coordinates Analysis (PCoA) based on weighted UniFrac Distances per year and sample type, for fungi **(A)** and for prokaryotes **(B)**; symbols represent sample types: Circles: Rhizosphere, Squares: Bulk soil, Triangles: Bare soil; **(C)** Venn diagram for rhizosphere specific fungal OTUs [fold change > 3 in case of rainy year (QS15-R); complete OTU table for rhizosphere dry year (QS16-R)]; **(D)** Venn diagram for rhizosphere specific prokaryotic OTUs (fold change > 3 in case of rainy year (QS15-R); complete OTU table for rhizosphere dry year QS16-R). Fold change > 3 is the rhizosphere OTUs divided by the sum of Bulk- and Bare- soil.

The beta-diversity analysis revealed that the precipitation conditions (rainy or dry) as well as the sample type influenced the community composition for fungi (ADONIS factor “year”: *R*^2^ = 0.329, *p* = 0.001; factor “sample type”: *R*^2^ = 0.281, *p* = 0.015) and prokaryotes (ADONIS factor “year”: *R*^2^ = 0.413, *p* = 0.001, factor “sample type”: *R*^2^ = 0.150, *p* = 0.01). In addition, the microbial community (fungi and prokaryotes) in the active rhizosphere clearly differed from all the other samples of both years (Figures 6A,B). Moreover, for prokaryotes, the bulk soil samples of the rainy year clustered with both, the dormant rhizosphere and bulk soil samples of the dry year. Whereas the fungal communities of the dry year grouped together (dormant rhizosphere, bulk, and bare soil). Finally, the fungal community of the bulk soil in the rainy year also visibly differed from all other samples.

#### Microbial Community Dynamics in Time

During the rainy year a total of 254 fungal OTUs was detected in all sample types (rhizosphere, bulk soil, and bare soil), raising to 397 OTUs in the dry year. Additionally, the proportion of OTUs shared between the rhizosphere and bulk soil samples increased from year 2015 to 2016 (Supplementary Figures S8a,b). In contrast, the sum of prokaryotic OTUs identified among all sample types was similar between the rainy and dry year (Supplementary Figures S8c,d). Interestingly, the proportion of OTUs shared between all samples increased, whereas the OTUs shared between the rhizosphere and bulk soil samples remained almost unchanged.

The microbial community changed drastically between the active and dormant rhizosphere. For comparison, in the active rhizosphere only the specifically enriched OTUs were considered (Supplementary Tables S18, S19), while in the dormant rhizosphere all OTUs were taken into account (Supplementary Tables S20, S21). Five fungal and 52 bacterial OTUs were shared between both years (Figures 6C,D and Supplementary Tables S22, S23). These values corresponded to 20.8% of the specific fungi and 32.3% of the specific prokaryotic community registered in the active rhizosphere; the most abundant OTUs were 2 unidentified and 1 corresponding to *Chaetomium undulatulum* among fungi (Supplementary Table S22), and OTUs corresponding to *Promicromonospora*, *Algoriphagus*, *Mesorhizobium*, *Altererythrobacter* and *Luteimonas* among bacteria, which strongly decreased in abundance in the dry year (between −55 and −99.9%). Additionally, in the active rhizosphere during the rainy year, taxa associated with plant growth promotion were detected, such as *Pseudomonas*, *Bacillus*, *Mesorhizobium*, *Mycobacterium*, *Neorhizobium*, and *Rhizobium* sp.

## Discussion

The Atacama Desert is known for its extreme aridity, where water availability is highly variable in time and space, occurring in specific, irregular and infrequent events (Chesson et al., 2004; Bull et al., 2016). The fleeting and biological context of the desert bloom phenomenon provide an interesting setting to study rhizospheric plant–microorganism interactions. Using as a model *C. longiscapa* populations in their natural habitat, we studied the dynamics of the rhizospheric microbiota along a latitudinal gradient, as well as between a rainy and a dry year.

### Microbiome Dynamics Along the Latitudinal Gradient

The overall most abundant fungal and prokaryotic phyla were *Ascomycota* and *Actinobacteria*, respectively, consistent with other studies of desert soils (Fierer et al., 2012; Makhalanyane et al., 2015). Other important prokaryotic phyla are *Proteobacteria* and *Chloroflexi*, especially the latter are typically in desert soils (Fierer and Jackson, 2006; Fierer et al., 2012).

The environmental conditions along the latitudinal gradient clearly shaped the microbial community in both, composition and diversity. We found that diversity and abundance of the fungal taxa evidently decreased with aridity, along the gradient (Maestre et al., 2015). Other soil parameters, as pH, organic matter and P-content, influenced mainly the fungal community structure (Tedersoo et al., 2014; Van der Walt et al., 2016; Wang et al., 2017). The observed variation of prokaryotic community could not be explained by the evaluated soil parameters.

In addition, *C. longiscapa* modifies soil conditions in the rhizosphere, generating a distinct microbial community (Lundberg et al., 2012; Lareen et al., 2016). We observed an overall lower diversity in the rhizosphere community than in the bulk soil, probably because a smaller number of microorganisms is selected by the rhizosphere (Hartmann et al., 2009; Fonseca-García et al., 2016).

Along the gradient, the rhizospheric fungal community was highly variable, sharing only 8.4% of the OTUs. These findings reflect the sensibility of soil fungi to changing climatic and edaphic conditions (Tedersoo et al., 2014). In contrast, rhizospheric bacterial communities had 44% of the OTUs in common, in disagreement other studies, which state that geographical distances reduce the similarity of bacterial communities (Martiny et al., 2011; Wang et al., 2017; Na et al., 2018). However, our results agree with those of Van der Walt et al. (2016) from the Namib Desert, suggesting that there may be “similar underlying drivers” for microbial distribution in both west coast deserts. Apparently, plants benefit more from bacterial associations under such extreme conditions than from fungi. Vikram et al. (2016) indicates some taxa found in our study (e.g., *Proteobacteria*, *Actinomycetales*, *Rhizobiales*, *Sphingomonadales*, and *Rubrobacterales*) as important in nutrient cycles in the desert by incorporating nitrogen, phosphorus, potassium, organic matter, among others, into the soil.

### Searching for the “Core Microbiome”

The “core microbiome” is a fraction of the rhizospheric community, which remains consistent at *C. longiscapa* roots independent from its developmental stages or soil types and fulfils important ecosystem functions (Colin et al., 2017; Pfeiffer et al., 2017). In this study, we identified a small rhizospheric core microbiome, which consists of 1 fungal and 5 prokaryotic OTUs.

The fungal OTU is *Fusarium concentricum*, representing 5.1% of the sequences obtained. Although it has been described as phytopathogen in sweet pepper (Wang et al., 2013), it is not necessarily prejudicial for native plants such as *C. longiscapa* (Lofgren et al., 2018; Selosse et al., 2018). The shared 5 prokaryotic OTUs represented 1.5% of sequences. Although these OTUs are known to interact with plants (e.g., *Ensifer* sp. and *Mesorhizobium* sp.), their presence in the rhizosphere is low compared to other studies (Colin et al., 2017; Hamonts et al., 2018). Nonetheless, they could contribute significantly to establishment of the rhizosphere community, as demonstrated by Dawson et al. (2017).

A cause for the minor size of the core microbiome are probably the divergent habitat conditions (soil physicochemical and environmental factors) along the latitudinal gradient (Nuccio et al., 2016; Na et al., 2018).

Moreover, we identified a small number of fungal and prokaryotic OTUs that were exclusive to the rhizosphere of each locality. Given their absence in the bulk soil, these taxa could be vertically transmitted by seeds. Considering that most seeds of annual desert plants are dispersed very little (Venable et al., 2008), this could explain their absence in the other localities. Nonetheless, these microorganisms could locally enhance the growth of *C. longiscapa.* For example, in Quebrada Seca 9 of these rhizosphere-exclusive OTUs were *Pseudomonas* sp., a genus known for its high plant growth promotion potential (Babalola, 2010).

### Co-occurrence Network

The analysis of co-occurrence patterns is a statistical approach to infer possible microbe-microbe interactions among complex microbiomes (Barberán et al., 2012). The method has been largely improved to obtain robust statistics, reduce false positives and limit habitat-filtering and compositionality (Faust et al., 2012; Berry and Widder, 2014; Weiss et al., 2016). Here, we detected three main clusters of correlated taxa, which likely represent recurrent microbial consortia. Interestingly, these three clusters not only included different taxa at Kingdom level (only bacteria, only fungi, or both), but were also habitat- and site-specific. The less abundant was the bacterial cluster, which was however present in all sites, following the gradient. The other two clusters appeared much more site-specific and were dominant. This general picture of the organization and distribution of microbial consortia highlights the strong effect of the desert habitat over the soil- and rhizosphere-microbiome, coherently with Marasco et al. (2018). However, the bacterial cluster appears to be consistently associated to *C. longiscapa*: the site-independent enrichment of this cluster in the rhizosphere further supports this conclusion. This cluster included five interacting phyla but was dominated by spore-forming *Actinobacteria*, which can survive under extreme drought, possibly creating the scaffold of the cluster. When the plant germinates during the rain events, the already existing consortium grows further in the rhizosphere, exerting beneficial effects on the plant. On the other hand, the more diverse mixed cluster is specifically associated to the less harsh site with more permissive environmental conditions.

### Microbiome Dynamics Between a Rainy and a Dry Year

Plants and microorganisms establish interactions in a highly specific manner, depending on soil type, season, plant phenology and genotype. These interactions are strengthened in arid and semi-arid environments, due to the harshness of the environment, facilitating the reciprocal exchange of metabolites to compensate for metabolic deficiencies (Taketani et al., 2017; Hassani et al., 2018). Our study included two contrasting seasons to determine the fluctuations in the resident microbiota between an active (rainy year 2015) and a dormant (dry year 2016) rhizosphere of *C. longiscapa* in the Quebrada Seca, the site with highest aridity.

During the rainy year, *C. longiscapa* recruits and selects through sugar-rich exudates, amino acids and organic acids specific microorganisms (Marasco et al., 2018), which could favor its development under different aridity conditions, in addition to repel others. Consistent with previous studies of arid environments, *Ascomycota* (fungi), *Proteobacteria* and *Bacteroidetes* (prokaryotes) were the most abundant phylum in the active rhizosphere (Maestre et al., 2015; Hassani et al., 2018). During the dry year, their abundance decreases between 16.4 and 27%, whereas the absence of a selective pressure by the rhizosphere promotes other microorganisms, e.g., *Actinobacteria, Chloroflexi* and *Firmicutes* (increase by 9.3 to 14%).

The active rhizosphere contained less fungal OTUs than the dormant rhizosphere, whereas OTUs shared among all types of soil increase in the dry year. These results suggest that once the plant dies, a reestablishment of a common soil microbiota occurs, including a colonization of the phyla present in the bulk soil previously excluded from the rhizosphere by the plant. Regarding the prokaryotes, although the number of OTUs did not change significantly between the years, an increase in the shared OTUs between the dormant rhizosphere and the bulk soil occurred, reinforcing the concept of soil recolonization. Our results highlight the control of the rhizosphere over microbial diversity, while at the same time suggesting a resilience of the soil microbiota of Quebrada Seca to recover from disturbances, like the selection exerted by the rhizosphere (Hartmann et al., 2009; Vargas-Gastélum et al., 2015; Fonseca-García et al., 2016).

The active rhizosphere displayed significant differences in the microbial community structure compared to all other samples, underlining the importance of the plant influence, coherently with other studies (Lareen et al., 2016; Dawson et al., 2017; Hamonts et al., 2018). Twenty-four fungal and 161 prokaryotic OTUs were specifically enriched in the active rhizosphere, suggesting that only a small fraction of the microbial soil diversity can be selected by the plant and/or be involved in mutualistic interactions (Hartmann et al., 2009; Dawson et al., 2017). Moreover, the proportion of shared OTUs between the active and dormant rhizosphere is low (57 OTUs) and abundance of those OTUs is noticeably reduced during the dry year. After rainfall, a quick plant establishment is triggered and a specific microbiome is recruited in the rhizosphere of *C. longiscapa* from the existing soil microbial diversity (Hartmann et al., 2009; Hassani et al., 2018). This specific community apparently fulfills a functional role in the rhizosphere of *C. longiscapa*, contributing to its optimal development and ensuring the offspring of future generations. Based on our results, the soil microbiota of the Atacama Desert is very dynamic and adaptable in short periods of time, re-establishing an apparently long-term stable community after plant’s death.

Additional studies involving different years of the desert bloom phenomenon are required to better understand the dynamics of the microbiota associated with *C. longiscapa* in the long-term, as well as the stability of bulk and bare soil during rainy and dry years. In addition, seasonal factors could influence these communities of microorganisms and the colonization of *C. longiscapa* at the time of germination in the next desert bloom should be determined.

Returning to our initial hypothesis, we found that fungal and bacterial communities respond differently to environmental conditions. The diversity and richness of fungal OTUs were negatively correlated with aridity, as predicted. However, interestingly, the community structure was predominantly influenced by other soil characteristics (pH, organic matter content) but not by aridity. In contrast, diversity, composition and structure of the bacterial community were not influenced by aridity or any other evaluated soil parameter. These findings coincide with the identification of site-specific microbial communities, not shared along the sites and of which ecological functions and dispersal mechanisms are unclear. In the rhizosphere of *C. longiscapa* the microbiota clearly differs in composition and structure from the surrounding bulk soil. A core microbiome is described, whose ecological function and interaction with the plant remain uncertain and should be studied in future.

In context of plant–microbe interactions in desert environments, our study contributes new insights regarding the importance of aridity in microbial community structure and composition, discovering the influence of other soil parameters in this complex dynamic network, which needs further to be investigated.

## Data Availability Statement

The sequences were deposited in the NCBI database (https://www.ncbi.nlm.nih.gov) in four BioProjects to facilitate the download of the information separately. In the BioProjects PRJNA561568 and PRJNA561410 there are the libraries for analyses between sites, for fungi and bacteria, respectively. In the PRJNA561612 and PRJNA561600 BioProjects there are the libraries for analyses between rainy and dry year for fungi and bacteria, respectively.

## Author Contributions

AS and MC designed the research. AS and JA sampled and performed laboratory work under the guidance of MC and SS. JA, MG, and MC carried out data analysis. All authors wrote and revised the manuscript, designed tables and figures, and approved the final version.

## Conflict of Interest

The authors declare that the research was conducted in the absence of any commercial or financial relationships that could be construed as a potential conflict of interest.

## Footnotes

1 http://qiime.org/home_static/dataFiles.html

2 https://www.ncbi.nlm.nih.gov

3 http://bioinformatics.psb.ugent.be/webtools/Venn/ebtools/Venn/
