# Supplementary material for: Microbiome Dynamics Associated With the Atacama Flowering Desert
Source: Front Microbiol. 2020 Jan 22;10:3160. doi: 10.3389/fmicb.2019.03160 (PMC6990129; doi:10.3389/fmicb.2019.03160)
Supplement: Supplementary file 1 [file Image_1.pdf]

Supplementary figures

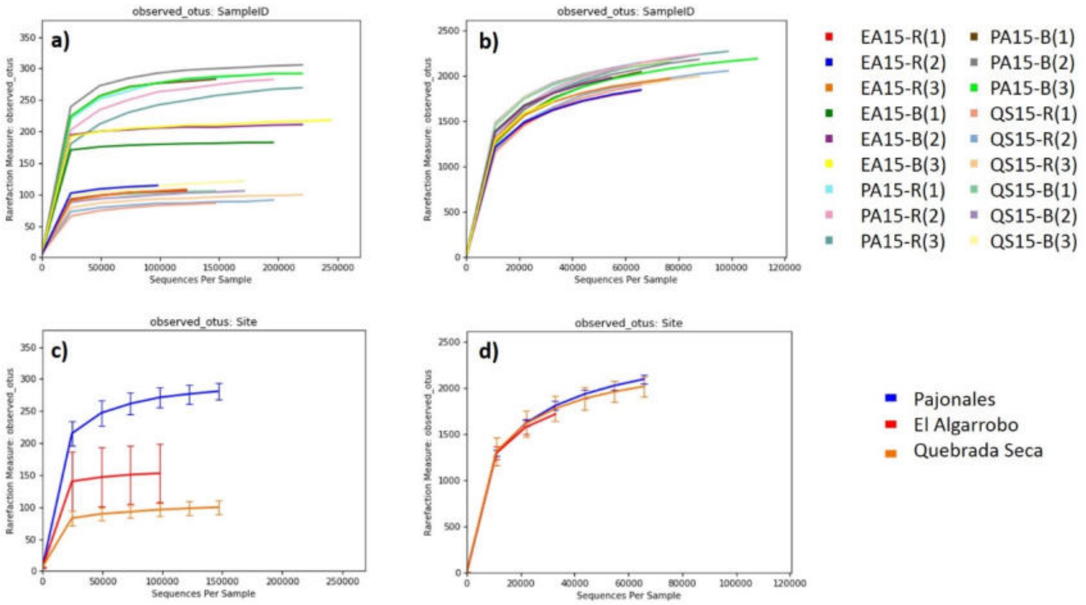

Figure S1: Rarefaction curves: analysis per sample type and locality. a) N. of fungal OTUs per sample; b) N. of prokaryotic OTUs per sample; c) N. of fungal OTUs per locality; d) N. of prokaryotic OTUs per locality.

Abbr. of sampling sites: QS – Quebrada Seca, EA – El Algarrobo, PA – Pajonales

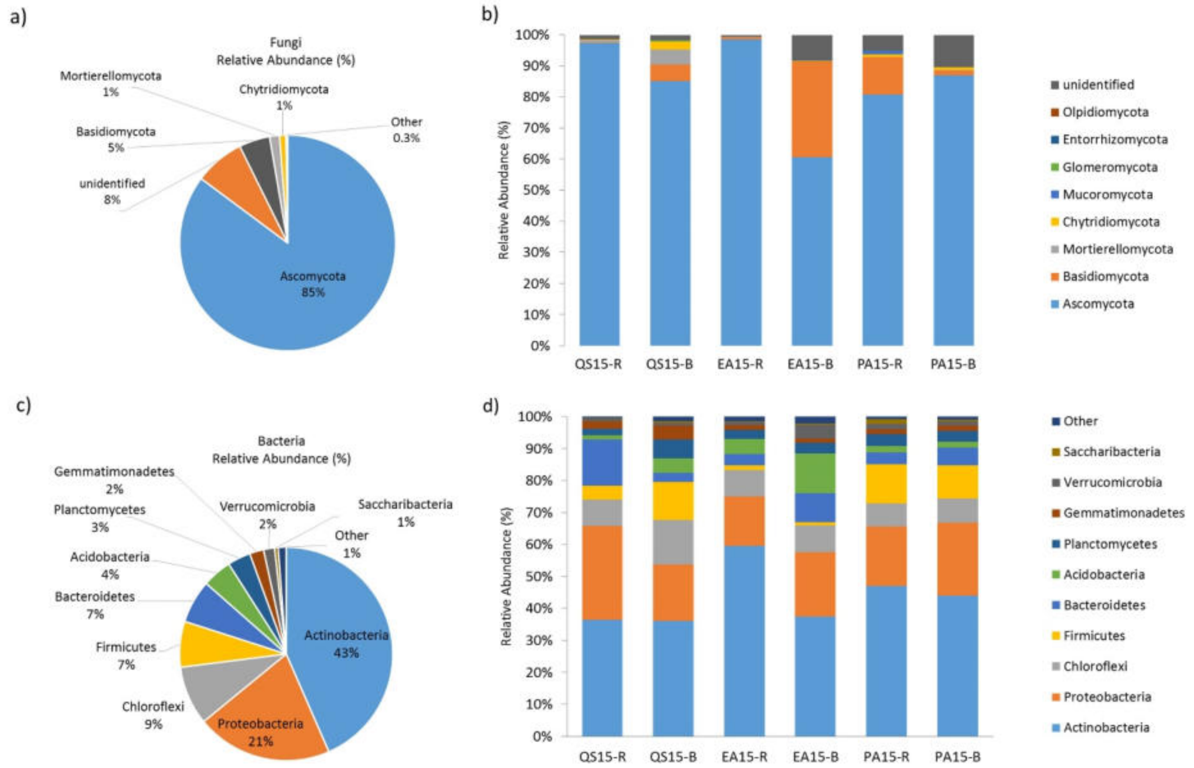

Figure S2: Taxonomic composition and relative abundance (%) at phylum level: analysis per locality and sample type (rhizosphere (R) and bulk soil (B)). a) Total relative abundance fungi (n=18); b) Relative abundance for fungi as average per locality and sample type (n=3); c) Total relative abundance prokaryotes (n=18); d) Relative abundance for prokaryotes as average per locality and sample type (n=3). Relative abundance shown in b and d corresponding to ≥ 0.5 %. Abbr. of sampling sites: QS – Quebrada Seca, EA – El Algarrobo, PA – Pajonales

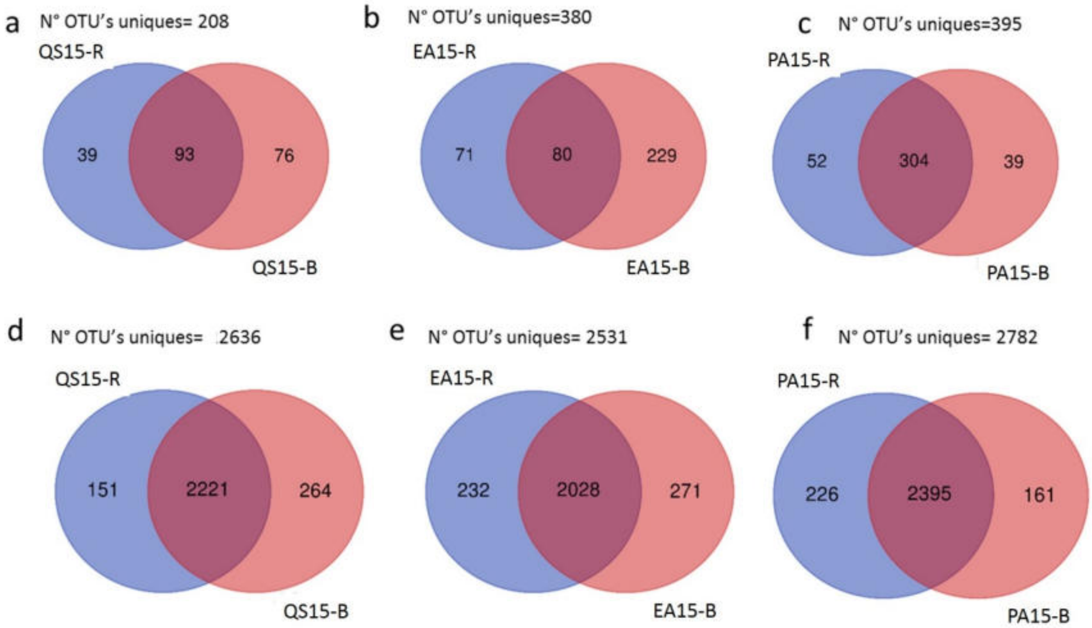

Figure S3: Venn diagrams comparing the number of exclusive OTUs of rhizosphere and bulk soil samples. a) fungi, locality Quebrada Seca (QS); b) fungi, locality El Algarrobo (EA); c) fungi, locality Pajonales (PA); d) procaryotes, locality Quebrada Seca; e) procaryotes, locality El Algarrobo; f): procaryotes, locality Pajonales.

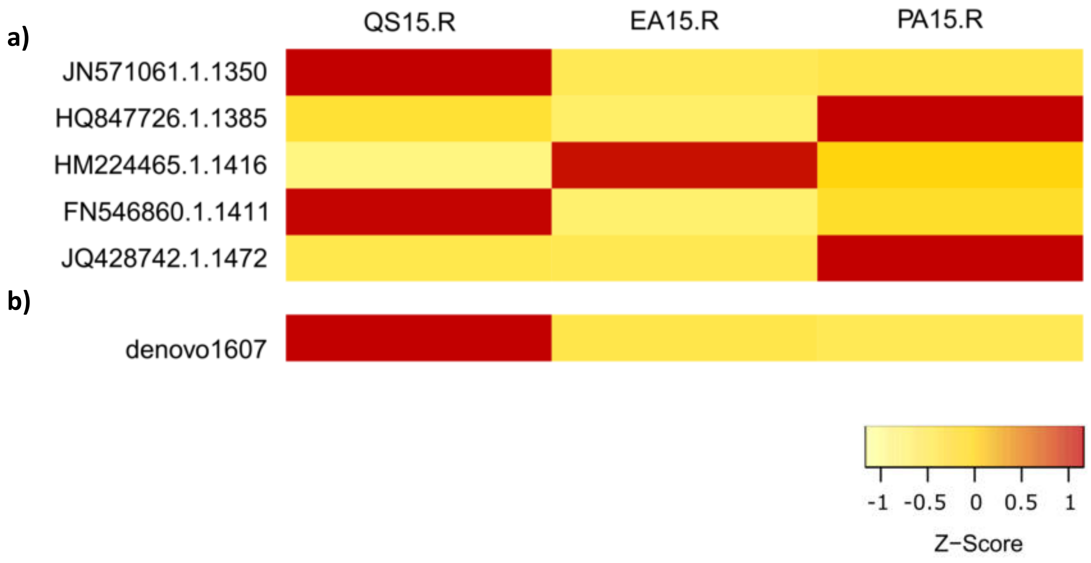

Figure S4: Heatmap of relative abundance for the core microbiome. a) prokaryotes; b) fungi. Abbr. of sampling sites: QS15.R – Rhizosphere at Quebrada Seca (year 2015), EA15.R – Rhizosphere at El Algarrobo (year 2015), PA15.R – Rhizosphere at Pajonales (year 2015).

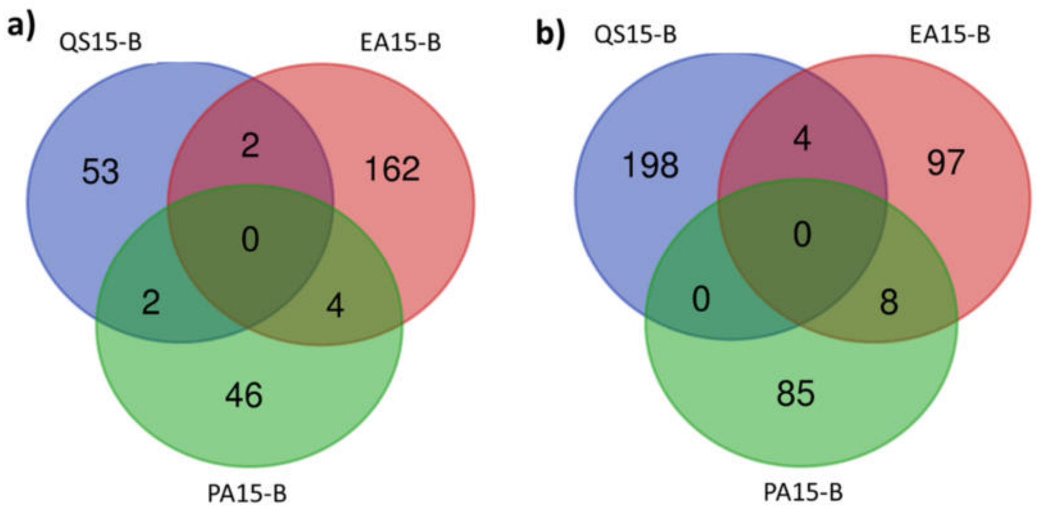

Figure S5: Venn diagrams comparing the number OTUs enriched in the bulk soil (B) between the localities. a) fungi; b) prokaryotes.

Abbr. of sampling sites: QS – Quebrada Seca, EA – El Algarrobo, PA – Pajonales

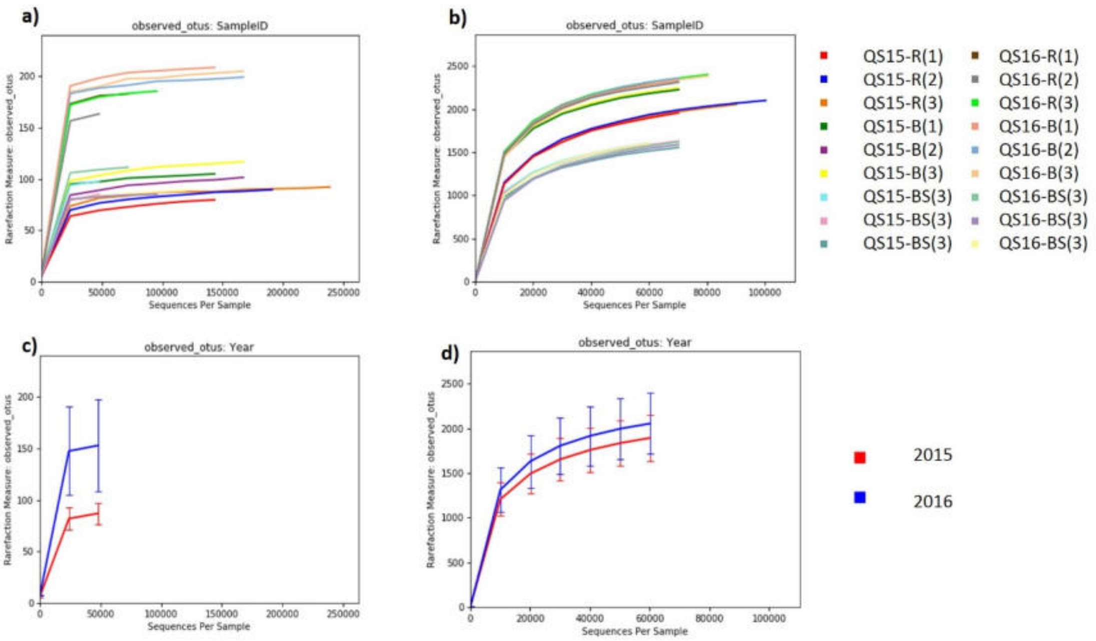

Figure S6: Rarefaction curves: analysis per sample type and year at Quebrada Seca (QS). a) Fungi Sample ID; b) prokaryotes Sample ID; c) N. of fungal OTUs per year; d) N. of prokaryotic OTUs per year.

Abbr. of sample types: rhizosphere (R), bulk soil (B), bare soil (BS)

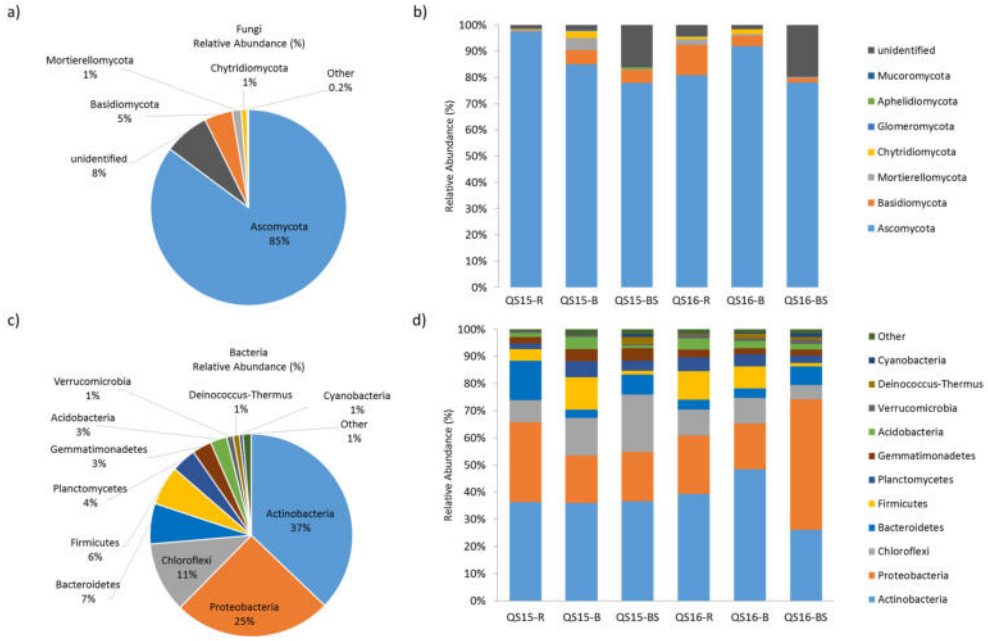

Figure S7: Taxonomic composition and relative abundance (%) at phylum level: comparison between rainy and dry year at Quebrada Seca (QS; rhizosphere (R), bulk soil (B), bare soil (BS)). a) Total relative abundance fungi (n=18); b) Relative abundance for fungi as average per locality and sample type (n=3); c) Total relative abundance prokaryotes (n=18); d) Relative abundance for prokaryotes as average per locality and sample type (n=3). Relative abundance shown in b and d corresponding to ≥ 0.5 %.

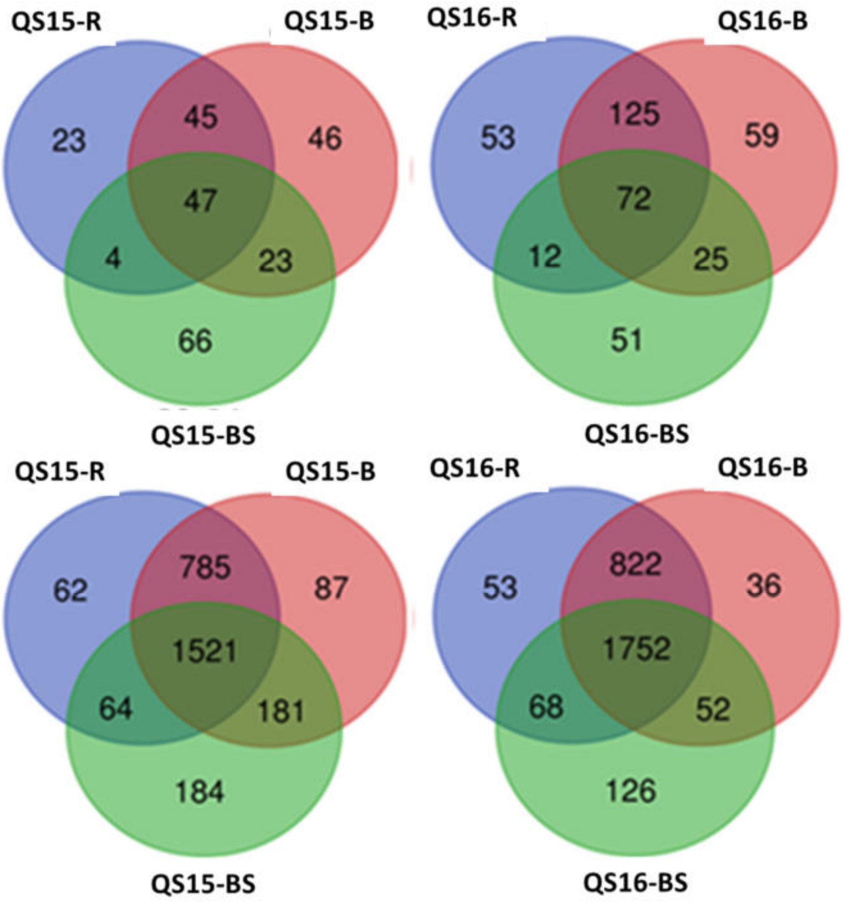

Figure S8: Venn diagrams comparing the number OTUs per sample type and year Quebrada Seca (QS). a) fungi rainy year (2015); b) fungi dry year (2016); c) prokaryotes rainy year (2015); d) prokaryotes dry year (2016).

Abbr. of sample types: bulk soil (B), bare soil (BS)
